# Impairment of Colour Vision in Diabetes with No Retinopathy: Sankara Nethralaya Diabetic Retinopathy Epidemiology and Molecular Genetics Study (SNDREAMS- II, Report 3)

**DOI:** 10.1371/journal.pone.0129391

**Published:** 2015-06-08

**Authors:** Laxmi Gella, Rajiv Raman, Vaitheeswaran Kulothungan, Swakshyar Saumya Pal, Suganeswari Ganesan, Tarun Sharma

**Affiliations:** 1 Elite School of Optometry, Chennai, Tamil Nadu, India; 2 Birla Institute of Technology and Science, Pilani, India; 3 Shri Bhagwan Mahavir Vitreoretinal Services, Sankara Nethralaya, Chennai, Tamil Nadu, India; 4 Manonmaniam Sundaranar University, Tirunelveli, Tamilnadu, India; Medical College of Soochow University, CHINA

## Abstract

**Purpose:**

To assess impairment of colour vision in type 2 diabetics with no diabetic retinopathy and elucidate associated risk factors in a population-based cross-sectional study.

**Methods:**

This is part of Sankara Nethralaya Diabetic Retinopathy Epidemiology and Molecular-genetics Study (SN-DREAMS II) which was conducted between 2007–2010. FM 100 hue-test was performed in 253 subjects with no clinical evidence of diabetic retinopathy. All subjects underwent detailed ophthalmic evaluation including cataract grading using LOCS III and 45° 4-field stereoscopic fundus photography. Various ocular and systemic risk factors for impairment of colour vision (ICV) were assessed in subjects with diabetes but no retinopathy. P value of < 0.05 was considered statistically significant.

**Results:**

The mean age of the study sample was 57.08 ± 9.21 (range: 44–86 years). Gender adjusted prevalence of ICV among subjects with diabetes with no retinopathy was 39.5% (CI: 33.5–45.5). The mean total error score in the study sample was 197.77 ± 100 (range: 19–583). The risk factors for ICV in the study were women OR: 1.79 (1.00–3.18), increased resting heart rate OR: 1.04 (1.01–1.07) and increased intraocular pressure OR: 1.12 (1.00–1.24). Significant protective factor was serum high-density lipoprotein OR: 0.96 (0.93–0.99).

**Conclusions:**

Acquired ICV is an early indicator of neurodegenerative changes in the retina. ICV found in diabetic subjects without retinopathy may be of non-vascular etiology.

## Introduction

Various studies have demonstrated that individuals with diabetic retinopathy can present with impairment of colour (ICV) and that the severity of ICV increases with increase in the severity of retinopathy and presence of diabetic macular edema.[1–4] It is reported that ICV was associated with diabetic retinopathy, however the relationship between colour vision and diabetes mellitus with no retinopathy is not clearly defined.[2,5] Few studies have reported risk factors for ICV among subjects with diabetes.[1,6]

The aim of the present study was to report the prevalence of ICV among subjects with type 2 diabetes without any evidence of diabetic retinopathy in a population based study and to assess various associated systemic and ocular risk factors for ICV.

## Methods

The Sankara Nethralaya Diabetic Retinopathy Epidemiology and Molecular Genetic Study (SN-DREAMS II) was a follow-up study of SN-DREAMS I [7] which was conducted between 2007 and 2010. Among the 958 subjects followed in SN-DREAMS II, 253 subjects with no clinical evidence of diabetic retinopathy and who have undergone FM 100 hue-test were included for the current analysis. Subjects with best corrected visual acuity worse than 6/12, uncooperative subjects and subjects who were unable to understand the test procedure were further excluded from the study. We also excluded those subjects with any congenital colour blindness, history of any ophthalmological disease, advanced cataract and any chronic disease not associated with diabetes that could affect the visual system and also subjects who received any laser photocoagulation. The Ethics Committee and Institutional Review Board of Vision Research Foundation approved the study, and written informed consent was obtained from all subjects in accordance with the Helsinki Declaration.

A detailed history, including data on demographics, systemic and ocular history, was obtained from all patients. Body mass index (BMI) was calculated by using the formula: weight (in kilograms)/height (in meters)^2^. Waist circumference was measured with the hip taken as the greatest circumference (the widest protrusion of the hip) on both the sides and measurements were made to the nearest centimeter. Hypertension was defined as systolic blood pressure ≥140 mmHg or diastolic blood pressure ≥90 mmHg or history of use of antihypertensive medications. Resting heart rate per minute was taken after making the subject remain in a seated position for at least five minutes. The mean ocular perfusion pressure [8] was calculated using the formula $MOPP=\frac{2}{3}\left[DBP+\frac{1}{3}\;\left(SBP-DBP\right)\right]-IOP$

Biochemical investigations (total serum cholesterol, high-density lipoproteins, serum triglycerides, hemoglobin, glycosylated hemoglobin HbA1c) were conducted at the base hospital with the blood samples being collected from the subjects in a state of fasting. The low-density lipoprotein was calculated using the modified Friedewald formula for Indian population.[9] Anemia was defined as a hemoglobin concentration of <13 g/dl in men and <12 g/dl in women.[10] The subject was considered normoalbuminuric if Albumin Creatinine Ratio (ACR) was less than 30 mg/g, microalbuminuric if the ACR was between 30 and 300 mg/g, and macroalbuminuric if the ACR was above 300 mg/g respectively.[11]

Diabetic neuropathy assessment was done by measuring VPT using sensitometer (Dhansai Laboratory, India). The VPT was measured by a single observer by placing biothesiometer probe perpendicular to the distal plantar surface of the great toe of both legs. The VPT was measured at the voltage level when the subject reported the first sensation of vibration. The mean VPT measure of three readings of both legs was considered for the analysis. Diabetic neuropathy was considered as present if the VPT value was >20 V.[12]

All the subjects underwent comprehensive eye examination, including fundus evaluation which was conducted by 2 ophthalmologists. The grading of lens opacity was done according to the Lens Opacity Classification System (LOCS) III system. The severity of the lens opacities, according to photographic standards, was separated into four major groups: nuclear opalescence (NO), nuclear colour (NC), cortical (CC), and posterior subcapsular (PSC). Intergrader agreement was determined by having both graders assess the eyes of 50 subjects, recruited from the pilot study, who had various grades of cataract. The grading agreements were: NO (k = 0.87), NC (k = 0.83), CC (k = 0.89), and PSC (k = 0.81). The overall overage of grading agreement was high (k = 0.85).[13] A significant NC was identified by the presence of an LOCS III score of >4 for NO or >4 for NC. Similarly, a significant cortical cataract (CC) was identified by an LOCS III score of >2 for CC, and a significant posterior subcapsular cataract (PSC) was identified by an LOCS III score of >2.[14,15]

Colour discrimination was assessed monocularly with the Farnsworth-Munsell 100 (FM 100) Hue test, under the FM 100 hue viewing booth lighting condition (developed by K Zahiruddin et al [16]) at a distance of 30 cm with near correction in place. To make the subjects understand 100-Hue test and also to remove the effect of learning curve, we prepared a demonstration video for 100-Hue test which made the task understandable for the subjects and also allowed the subjects to perform test prior to the actual test of which the results are considered for the study. The FM 100-Hue test results were analyzed using web-based scoring software designed by Torok B **(http://www.torok.info/colorvision/dir_for_use.htm)**. Total error score (TES) was assessed based on the classical method. We defined subjects with Impaired Colour Vision (ICV) based on the criteria that if the total error score based on classical method fell outside the 95^th^ percentile for age as published by Verriest et al [17] for monocular testing without previous binocular experience and normal otherwise. Contrast sensitivity was assessed using Pelli-robson chart at a distance of 1 m and values were represented in log units. Intraocular pressure was assessed using Goldmann applanation tonometer (Zeiss AT 030 Applanation Tonometer, Carl Zeiss, Jena, Germany)

Retinal thickness was measured using Spectral domain optical coherence tomography (SD‑OCT) (Copernicus, Optopol, Poland), following pupil dilation with 1% tropicamide. SD-OCT was done using Asterisk scan protocol (7mm length scan, 6 B-scans with 2743 A-scans/B-scan). Using the computer-based caliper measurement tool in the SD-OCT system, the central foveal thickness was measured as the distance between the vitreo-retinal interface and the inner edge of the retinal pigment epithelium (RPE). The photoreceptor layer thickness was measured as the distance between the external limiting membrane (ELM), and the inner edge of the RPE. Mean retinal thickness in the central 1mm area was assessed.

Microperimetry (MP1, Nidek Technologies, Padova, Italy) was performed in the mydriatic state using Goldmann size III stimuli, 4–2 threshold strategy, and a white background with an intensity of four apostilbs. An automated program was used using 33 stimulus points, which were projected in the central 20° of fundus. The mean retinal sensitivity was expressed in decibels.

### Statistical Analysis

All statistical analyses were performed using the statistical software (SPSS for Windows, ver.15.0 SPSS Science, Chicago, IL). The data was checked for its distribution using one-sample Kolmogorov-smirnov test and found that the data was normally distributed. The results were expressed as mean ± SD if the variables were continuous and as percentages if categorical. The student t test for comparing continuous variables and the chi-square test for comparing proportions among groups were used. Multiple logistic regression analysis was used to assess the risk factors. p value of < 0.05 was considered statistically significant.

## Results

The mean age of the study sample was 57.08 ± 9.21 (range: 44–86 years). Gender adjusted prevalence of ICV among subjects with diabetes having no retinopathy was 39.5% (CI: 33.5–45.5). Significant difference in the mean TES between ICV and no ICV groups was found (287.01 ± 81.04 vs 139.45 ± 60.32; *p*< 0.001). Table 1 presents various systemic risk factors for ICV among diabetic subjects with no retinopathy. There was a significant difference in age between ICV and no ICV groups (*p* = 0.039). The duration of diabetes was found to be high in the no ICV group (8.3 ± 5.4 years) compared to the ICV group (7.1 ± 3.9 years) with border line significance (*p* = 0.054) and resting heart rate was found to be significantly high in ICV group (77.9 ± 10.4) compared to no ICV group (73.7 ± 9.6) with *p* = 0.001. Biochemical parameters like glycosylated hemoglobin, serum total cholesterol, serum triglycerides, and serum low-density lipoprotein did not show any association with prevalence of ICV except for high-density lipoprotein which was significantly low in ICV (35.78 ± 8.8 in ICV vs 38.5 ± 11.1 mg/dl in no ICV; *p* = 0.034). Other parameters like presence of hypertension, mean ocular perfusion pressure, BMI, nephropathy and presence of neuropathy did not show any association with ICV.

**Table 1:** Systemic risk factors associated with impaired color vision in diabetes without retinopathy.

Risk factors		No ICV	ICV	
	N = 253	153	100	*P*
Age (years)	253	58.0±9.4	55.6±8.8	**0.039**
Gender				
Men	149 (58.9)	97 (63.4)	52 (52.0)	0.072
Women	104	56 (36.6)	48 (48.0)	
Duration of Diabetes	253	8.3±5.4	7.1±3.9	0.054
HbA1c	253	7.2±1.8	7.4±1.8	0.501
Haemoglobin	253	13.9±2.1	13.9±1.7	0.987
Anemia				
Absent	206	121 (79.1)	85 (85.0)	0.237
Present	47	32 (20.9)	15 (15.0)	
Lipid Profile				
Serum total chlesterol	253	173.9±44.4	173.9±45.4	0.984
Serum HDL lipoprotein	253	38.5±11.1	35.78±8.8	**0.034**
Serum triglycerides	253	132.7±93.5	135.9±111.3	0.865
Serum low-density lipoprotein	253	104.7±35.2	104.2±41.3	0.918
Hypertension				
Absent	219	133 (86.9)	86 (86.0)	0.832
Present	34	20 (13.1)	14 (14.0)	
Resting heart rate	253	73.7±9.6	77.9±10.4	**0.001**
Mean ocular perfusion pressure	253	49.5±7.7	48.9±8.2	0.547
BMI (kg/m2)	253	25.6±5.7	25.7±7.5	0.935
Waist circumference	253	92.9±11.3	92.9±11.5	0.992
**Nephropathy**				
Normal	205	124 (81.0)	81 (81.0)	0.982
Micro	41	25 (16.3)	16 (16.0)	
Macro	7	4 (2.6)	3 (3.0)	
**Neuropathy**				
Absent	205	123 (80.4)	82 (82.0)	0.750
Present	48	30 (19.6)	18 (18.0)	

ICV:Impaired colour vision; HbA1c: Glycosylated hemoglobin; BMI: Body mass index; HDL: High density lipoprotein; LDL: Low density lipoprotein

Table 2 shows various ocular risk factors for ICV in subjects with diabetes without retinopathy. Significant ocular risk factors included subjects who had not undergone cataract surgery (no cataract surgery ICV 90% Vs those who had previously undergone cataract surgery ICV 10%; *p* = 0.023) and intraocular pressure which was significantly high in the group with ICV (14.7 ± 3.1 in ICV Vs 13.9 ± 2.3 in no ICV; *p* = 0.03).

**Table 2:** Ocular risk factors associated with impaired colour vision.

Risk factors		No ICV	ICV	
	N = 253	153	100	*P*
**Present of cataract (LOCS III grade)**				
No Cataract	195	113 (93.4)	82 (91.1)	0.537
Any Cataract	16	8 (6.6)	8 (8.9)	
Monotype				
NC	1	1 (0.8)	0 (0)	1.000#
CC	8	5 (3.3)	3 (1.9)	1.000#
PSC	5	1 (0.8)	4 (4.4)	0.166#
Mixed				
NC + CC	2	1 (0.7)	1 (1.0)	1.000#
NC + PSC	-	-	-	
CC + PSC	-	-	-	
NC+ CC + PSC	-	-	-	
**Cataract Surgery**				
No	211	121 (79.1)	90 (90.0)	**0.023**
Yes	42	32 (20.9)	10 (10.0)	
**Intraocular pressure**	239	13.9±2.3	14.7±3.1	**0.03**
**Contrast Sensitivity**	223	1.3±0.21	1.3±0.17	0.996
**OCT**				
Central retinal thickness (1mm)	195	184.6±29.8	187.9±32.7	0.481
Central foveal thickness	223	170.6±22.6	171.3±19.5	0.824
Photoreceptor layer thickness	223	60.7±7.4	60.7±5.4	0.968
**Mean retinal sensitivity**	160	14.9±3.0	15.0±3.4	0.77

NC: Nuclear cataract; CC: Cortical cataract; PSC: Posterior sub capsular cataract; OCT: Optical coherence tomography

# Fishers exact test

Table 3 presents multiple logistic regression analysis of risk factors for presence of ICV in our study population. After adjusting for all other associated factors, it was shown that women were at higher risk of developing ICV (OR: 1.79, (1.00–3.18)). The duration of diabetes was no longer associated with ICV after adjusting for other factors. Increased intraocular pressure (OR: 1.12, (1.00–1.24)), resting heart rate (OR: 1.04, (1.01–1.07) were significant risk factors. Undergoing cataract surgery (OR: 0.50, (0.28–0.91)) was a protective factor for ICV.

**Table 3:** Multiple logistic regression model for risk factors for Impaired colour vision in the study population.

Risk factors	CVI	
	OR (95% of CI)	*P*
Women	1.79 (1.00–3.18)	**0.049**
Duration of Diabetes	0.95 (0.89–1.02)	0.139
Anemia present	0.85 (0.41–1.77)	0.661
Serum HDL lipoprotein	0.96 (0.93–0.99)	**0.011**
HbA1c	0.99 (0.85–1.16)	0.926
Resting heart rate	1.04 (1.01–1.07)	**0.005**
Cataract Surgery done	0.46 (0.19–1.09)	0.079
Intraocular pressure	1.12 (1.00–1.24)	**0.048**

Adjusted variables: Gender, Duration of diabetes, Anemia, Serum HDL lipoprotein, HbA1c, Resting heart rate, cataract surgery, intraocular pressure

## Discussion

Colour vision theory emphasizes several stages of visual processing: pre-receptoral filters (lens, macular pigment, pupil), cone photopigments (L-, M-, and S-cones) and post-receptoral processes (red-green, S-cone, and luminance channels). Previous studies have found a relationship between colour vision impairment and diabetic retinopathy [1–4] and also among subjects with diabetes without retinopathy [18]. Prevalence of ICV as reported by Shoji et al [6] was 3.5% in diabetic subjects without retinopathy. The prevalence of ICV in our study (39.5%) was higher compared to those reported earlier. Shoji et al [6] estimated ICV in two steps; Ishihara plates, a Lanthony 15-hue desaturated panel and Standard Pseudoisochromatic Plates Part 2 were used to examine colour vision and Farnsworth-Munsell 100-hue test was performed to define acquired colour vision impairment. Moreover, the study subjects were between 20–60 years, a predominantly younger population. This difference in the prevalence between our study and the other could be due to the difference in the study methodology and the sample population. To the best of our knowledge this is the first study from India to report the prevalence of ICV in diabetic subjects without retinopathy.

Women were at higher risk of developing ICV in the study sample which was not found in the earlier studies.[1, 17] It has been reported by Giuffre et al that the performance of FM 100-hue test improved for 10 out of 15 participants at the time of ovulation, as opposed to during menstruation or at the beginning of the cycle. [19] Eisner et al [20] have showed evidence suggesting that estrogenic response affects the colour naming of short-wavelength test stimuli presented on 580-nm backgrounds.

We observed that prevalence of ICV was significantly high among subjects who did not undergo cataract surgery. It is known that as the yellow chromophores accumulate inside the lens with increase in age it reduces the transmission of blue light to the retina which results in blue-yellow colour vision defects. Non-enzymatic glycation of lens proteins causes browning of the lens and that is known to be accelerated in diabetes. [21] Accelerated yellowing, rather than neuronal or vascular changes in the retina, thus appears to be the primary cause of the colour vision anomaly observed in subjects with diabetes.[22] Ventruba J [23] reported that colour vision was significantly improved in subjects following cataract surgery which supports to our results that undergoing cataract surgery is a protective factor.

Increased intraocular pressure was found to be a significant risk factor for ICV which was supported by previous studies.[24,25] This can be attributed to the fact that the short wavelength cones or their neuronal connections are less able to resist the raised intraocular pressure.[24] Further, the blue-yellow ganglion cells have a unique morphology and connectivity to second order neurons leading the blue-yellow ganglion cells to be more susceptible to IOP related damage.[25] The microvascular complications other than diabetic retinopathy and neuropathy did not show any association with ICV in diabetic subjects.

Acquired ICV is an early indicator of neurodegenerative symptoms. It has been suggested that neural damage possibly precedes clinical diabetic retinopathy. It has also been reported that the thickness of the retinal photoreceptor layer and retinal sensitivity were decreased in patients with diabetes, although no microvascular changes were noted in the retina.[26] On the contrary, in our study we did not find any further change in the SD-OCT thickness parameters and the mean retinal sensitivity of diabetic subjects having no retinopathy and with ICV. The evidence from this study supports the view that the ICV found in diabetic subjects without retinopathy may be of non-vascular etiology. Several hypotheses have been proposed such as osmotic distortion of the retina caused by fluid shifts inside the retina, followed by distortion and dysfunction of the neural cells and disorders of metabolism of neural cells caused by direct damage due to diabetes or because of the alterations of retinal microcirculation. The mechanism responsible for the ICV is yet to be fully clarified and further research on these pathogenic mechanisms is needed.

The limitation of our study being conducted in a specific diabetic population could not extrapolate the results to more general diabetic population as the sample size was calculated to estimate the prevalence of DR rather than for the ICV.

To conclude, ICV was noted even among diabetic subjects without retinopathy. Significant risk factors for ICV were female gender and increased intraocular pressure. Our study finding re-emphasizes the importance of colour vision assessment even among subjects with diabetes but no retinopathy, which will be a useful method of screening and monitoring the diabetic subjects even without retinopathy as colour vision plays an important role in subjects with diabetes to reliably self-monitor the blood and urine glucose levels.
